# Short-term effect of COVID-19 pandemic on cryptocurrency markets: A DCC-GARCH model analysis

**DOI:** 10.1016/j.heliyon.2023.e18847

**Published:** 2023-08-05

**Authors:** Kais Ben-Ahmed, Saliha Theiri, Naziha Kasraoui

**Affiliations:** a Department of Finance, College of Business, University of Jeddah, Saudi Arabia; b Higher Institute of Management, ISG, University of Sousse, Tunisia; c Faculty of Economic Sciences and Management of Tunis, Tunis El Manar University, El Manar, Tunisia

**Keywords:** Cryptocurrency, Volatility, DCC-GARCH models, Short-term effect, Panic coronavirus index, COVID-19

## Abstract

This research examines the impact of the coronavirus index on the returns and volatility of ten major cryptocurrencies during the COVID-19 pandemic. For this purpose, we applied a multivariate volatility GARCH model with an integrated dynamic conditional correlation (DCC) approach to daily cryptocurrency values observed data during the January-December, 2020 period. Moreover, we used the Granger causality test to study return-volume correlations. The findings indicate that cryptocurrency volatility declined after the World Health Organization declared on March 11, 2020, that the coronavirus was a pandemic. Unlike most of the relevant previous studies, we found that the COVID-19 crisis did not have a long-term effect on cryptocurrency returns and volatility but only presented a short-term effect. Our results have implications for investors who need to determine an optimal portfolio for a scenario other than the base.

## 1 Introduction

The covid-19 crisis has inspired a new stream of literature that treated the impact of the pandemic's impact on currency markets. The aim of this research is to support the current market's players and its regulators, with the intention of predicting the currencies' behavior during financial distress periods. Accordingly, financial markets worldwide have been smitten sharply by the recent COVID-19 pandemic that has fueled panic and anxiety among investors and shaped the first widespread bear market at the beginning of the cryptocurrency's transactions.

We have extensively reviewed studies that examined the impact of COVID-19 using several indexes and databases, including Web of Science, Scopus and papers published in English. A total of 28 papers were ultimately reviewed and included in this study. Most of them link COVID-19 data to the world stock markets. However, this study focuses on the impact of COVID-19 on the returns and volatility of ten major cryptocurrencies observed from January 1 to December 31, 2020. In addition, all COVID-19 studies of its impact on stock markets and cryptocurrencies returns are as up-to-date as possible and include the most recent studies covering the 2020-2022 period. Worth noting is that the main conclusions of these studies are similar to some extent, and there is high coherence between COVID-19 waves and cryptocurrencies returns.

Although further research needs to be done, over of the last three years many improvements have been made to the way the topic is apprehended. Specifically, epidemiological [15] and statistical [16] analyses were performed in the financial sector following COVID-19. Nonetheless, despite the substantial progress made by these studies, unfortunately, the reviewed findings are not, on the whole, very conclusive and some are even contradictory to recommend actions about the real impact of COVID-19 on cryptocurrencies returns.

The cryptocurrency markets represent a complex financial system. Traditional asset pricing models and common risk factors cannot explain cryptocurrency returns. Moreover, there is no data, like earnings, dividends and cash flows, essential to unravel cryptocurrencies' dynamic, making them unpredictable [15]. Therefore, in the last three years, numerous studies have been conducted on the possible links between COVID-19 and stock market returns [13], [22]. Specifically, the link between COVID-19 and a stock market performance has been examined by [21], [24] focusing on commodity markets, [17], [30], [31] cryptocurrency markets, and [18], [19] alternative investment markets. Moreover, other studies examined the impact of COVID-19 on the volatility cryptocurrency series [2]. The results obtained from these previous studies are often contradictory. In other words, cryptocurrency returns and volatilities in such diverse investment portfolios showed strong evidence of positive and significant dependence on COVID-19.

New studies have emerged highlighting the relationship between COVID-19 outbreak and major financial markets. Limited to the effect of COVID-19 on cryptocurrency returns, this literature reported mixed results. Umar and Gubareva (2020) and Naeem et al. (2021) showed that COVID-19 outbreak adversely affected the efficiency of cryptocurrency returns. The authors conclude that cryptocurrencies, which could be traded under normal market conditions, are likely to fail during the COVID-19 pandemic [25], [21]. Apergis (2021), using a group of eight major cryptocurrencies' returns, observed over the February 1, 2020, through October 31, 2021 period, found that cryptocurrency returns responded negatively to the COVID-19 pandemic [1]. Likewise, David (2021) founds that COVID-19 exerted a negative effect on cryptocurrencies during a short period of financial panic between March and April, 2020 [26]. However, the author also asserts that since April 2020, the cryptocurrency market progressively recovered its initial state because COVID-19 was continuously disappearing. Goodell (2020) showed that COVID-19 waves caused a massive rise in cryptocurrency prices, namely, Bitcoin [10]. By contrast, Corbet et al. (2020) found that Bitcoin did not act as a currency hedge or a safe-haven [5], [14].

Given the interconnections between financial markets, another stream of research has examined the impact of COVID-19 on long-term memory in returns and volatility of cryptocurrency and stock markets. Lahmiri and Bekiros (2021), examining 45 cryptocurrency markets and 16 international equity markets, found consistent evidence supporting the conclusion of a high level of persistent variability in return series in both digital currencies and stock markets. Using ARFIMA and FIGARCH models, they also assert that the COVID-19 pandemic significantly affected long-term memory in returns and volatility of cryptocurrency and international stock markets. Ultimately, they conclude that ARFIMA-FIGARCH models are significantly suitable to describe the returns and volatility of cryptocurrencies before and during the COVID-19 pandemic [20]. Moreover, other studies on the presence of long-term memory in the cryptocurrency market have been conducted. Specifically, Charfeddine et al. (2020) used several tests to study the relationship between long-term memory and market inefficiency for cryptocurrencies. They provide new evidence that confirms that cryptocurrency volatility has a long-term memory rather than sporadic shifts [7].

Bearing on the above studies, there is no conclusive results on the effects of COVID-19 on cryptocurrency returns. In addition, there is insufficient evidence to support such a relationship, despite the plethora of studies treating the issue. Moreover, the results are contradictory because the samples vary and the methods used are different from one study to another. Consequently, it would thus be of great interest to shed more light on the behavior of cryptocurrency returns during a crisis period, like the COVID-19 pandemic.

The objective of the present study was to examine the dynamic interconnections between COVID-19 and cryptocurrency markets. Indeed, studying the impact of COVID-19 on cryptocurrency markets is very relevant. This study (1) could be considered a reference study on the impact of the COVID-19 panic index on returns and volatilities of currencies, (2) could reveal differences in the responses of cryptocurrencies to the pandemic panic index, (3) could show the evolution of cryptocurrencies' values over time, and (4) could provide investors with practical information on asset allocation and portfolio management during the COVID-19 pandemic. In doing so, we applied a multivariate volatility GARCH model with an integrated dynamic conditional correlation (DCC) approach to daily cryptocurrency values observed data during the January-December, 2020 period. Furthermore, we show that cryptocurrency correlation responds to the Coronavirus Panic Index.

The remainder of this paper is structured as follows. Section 1 briefly reviews the relevant literature. Section 2 describes the data and outlines the empirical methodology. Section 3 presents and discusses the main empirical results. Section 4 concludes.

## 2 Methodology

### 2.1 Data

We examine the bivariate relationship between COVID-19 panic, measured by the Coronavirus Panic Index (PI), and cryptocurrencies (CM). To this end, we consider a group of ten major CM' returns, namely Cardano, Bitcoin, Dogecoin, Polkadot, Etherum, Litcoin, Tron, Uniswap, Stellar, and Ripple, and their volatility, observed from January 1, 2020, to December 31, 2020. We use daily data, since their transaction volume approximates 75% of total CM volume. We also use a new proxy for COVID-19, namely the coronavirus index obtained from Ravenpack. The index ranges between 0 and 100, with 0 implying the lowest level of coronavirus panic and 100 indicating the highest level. Table 1 shows the summary statistics for each variable from January–to December 2020. Table 1 indicates that volatility was primarily supported by XRP. Furthermore, as shown in Table 1, all CM exhibit skewness and excess kurtosis. Therefore, from the skewness metric, one might conclude that these CMs display an asymmetric distribution. In addition, these CMs show positive kurtosis coefficients indicating that the return series is peaked and thick tailed. This means that these CMs are not normally distributed and show a leptokurtic distribution, rejecting the normality property. Daily PI has positive kurtosis (leptokurtic distribution), which means it has a sharper peak and heavier tails than a normal distribution. The unit root test of ADF (Augmented Dickey-Fuller) shows that all the CMs are non-stationary at levels but turn into stationary at the first differences.

**Table 1:** Summary statistics and unit root test, January 1 to December 31, 2020.

CM	Mean	SD(St.Dev)	Max.	Min.	Skewness	Kurtosis	ADF–1(1)
ADA(*Cardano*)	0.0044	0.0623	0.2010	–0.5360	–1.6731	19.1105	−21.3508^⁎⁎⁎^
BTC(*Bitcoin*)	0.0039	0.0428	0.1459	–0.4973	–4.3929	56.7852	−21.8412^⁎⁎⁎^
DOGE(*Dogecoin*)	0.0025	0.0538	0.1746	–0.5813	–3.8099	43.1801	−21.5073^⁎⁎⁎^
DOT(*Polkadot*)	0.0043	0.0717	0.2600	–0.6371	–1.7845	21.9432	−20.60251^⁎⁎⁎^
ETH(*Etherum*)	0.0051	0.0571	0.2302	–0.5896	–3.0279	37.5221	−21.2210^⁎⁎⁎^
LTC(*Litcoin*)	0.0022	0.0556	0.2299	–0.4868	–1.8960	21.0484	−21.5611^⁎⁎⁎^
TRX(*Tron*)	–0.0003	0.0011	0.0101	–0.0065	1.3976	30.8617	−15.2403^⁎⁎⁎^
XLM(*Uniswap*)	0.0022	0.0622	0.3924	–0.4403	0.0055	15.9827	−7.3380^⁎⁎⁎^
UNI(*Stellar*)	0.0006	0.0225	0.0897	–0.1277	–0.8426	10.7959	−3.9213^⁎⁎⁎^
XRP(*Ripple*)	3.4000	0.0642	0.3397	–0.5410	–1.6308	26.5872	−12.3618^⁎⁎⁎^
PI(*Panic Index*)	–0.0038	0.6938	1.8845	–2.8679	–0.2191	4.7022	−9.3683^⁎⁎⁎^

Notes: ^⁎⁎⁎^ (^⁎⁎^/^⁎^) denote 1%, 5% and 10% level of significance respectively.

Source: Own calculations

### 2.2 Econometric framework

As the CM series and daily PI are asymmetric and leptokurtic, we consider that conditional volatility resulting from the Garch models is appropriate for investigating the connection between CM returns and PI. Moreover, the Dynamic Conditional Correlation Generalized Autoregressive Conditional Heteroskedasticity (DCC-GARCH) model, rather than the Constant Conditional Correlation GARCH (CCC-GARCH) model, investigates the dynamic correlation structure over time. Another benefit of this model is detecting possible changes in conditional correlation over time, allowing us to find out dynamic CM' behavior in response to the COVID-19 panic index. Furthermore, using the DCC-GARCH model, we have the advantage of (i) measuring the correlation coefficients of the residuals and (ii) accounting directly for heteroscedasticity [4]. Therefore, one might support that, unlike Forbes and Rigobon (2002), correlation for the time-varying volatility is continuously adjusted by the DCC–GARCH model [8]. Consequently, DCC captures well correlation [6].

Following Engel and Sheppard (2001), we assume that returns have conditionally multivariate normal distribution with zero expected values of returns $r_{t}^{2}$ and variance-covariance matrix $H_{t}$. Consequently, CM returns, given the information set available at time t−1 ($\xi_{t-1}$), have the following distribution:

$$r_{t}/\xi_{t-1}∼\text{N}(0,H_{t})$$

and

$$H_{t}\equiv D_{t}R_{t}D_{t}$$

$D_{t}$ represents a k×k diagonal matrix of time varying standard deviations from the univariate GARCH model. $R_{t}$ is the time varying correlation matrix.

$$D_{t}=diag(\sqrt{h_{11,t}},\ldots ,\sqrt{h_{kk,t}})$$

More specifically, parameters of the matrix $D_{t}$ are given by a univariate GARCH model (Engle and Sheppard 2001).

(1) $$h_{it}=\omega_{i}+\sum_{p=1}^{p_{i}}\alpha_{ip}r_{it-p}^{2}+\sum_{q=1}^{Q_{i}}\beta_{iq}h_{it-q}$$

for i=1,2,...,k (variables, in our case CM returns), with the usual GARCH restrictions (for non-negativity and stationarity $\sum_{p=1}^{p_{i}}\alpha_{ip}+\sum_{q=1}^{Q_{i}}\beta_{iq}<1)$.

$$R_{t}=diag(\sqrt{q_{11,t}},\ldots \sqrt{q_{kk,t}})Q_{t}diag(\sqrt{q_{11,t}},\ldots \sqrt{q_{kk,t}})$$

$Q_{t}$ is a symmetric positive definite matrix.

(2) $$Q_{t}=(1-\sum_{m=1}^{M}\alpha_{m}-\sum_{n=1}^{N}\beta_{n})\bar{Q}+\sum_{m=1}^{M}\alpha_{m}(\varepsilon_{t-m}\varepsilon_{t-m}^{\prime })+\sum_{n=1}^{N}\beta_{n}Q_{t-n}$$

$\bar{Q}$ is the unconditional covariance of the standardized residuals. The parameters $\alpha_{m}$ and $\beta_{n}$ are non-negative with $\sum_{p=1}^{p_{i}}\alpha_{ip}+\sum_{q=1}^{Q_{i}}\beta_{iq}<1$.

Lastly, the parameters of the multivariate DCC-GARCH models are estimated by the quasi-maximum likelihood method (QMLE) using Broyden, Fletcher, Goldfarb and Shanno's (BFGS) algorithm. The loglikelihood function is expressed as follows:

$$L=-\frac{1}{2}\sum_{t=1}^{T}(klog(2\pi )+2log(\left|D_{t}\right|)+log(\left|R_{t}\right|)+\varepsilon_{t}^{\prime }R_{t}^{-1}R_{t}\varepsilon_{t})$$

Where $\varepsilon_{t}∼\text{N}(0,R_{t})$ are the residuals standardized by their conditional standard deviation. The DCC-GARCH model is estimated in two stages. The first consists of estimating the univariate GARCH model, while the second involves measuring the time-varying conditional correlation.

In our empirical estimation, a bivariate DCC(1,1)-GARCH(1,1) model is estimated with ten CMs. The parameters of the DCC-GARCH model, *θ*, are written in two groups: $\left(\phi_{1},\phi_{2},\ldots ,\phi_{k},\psi )=(\phi ,\psi \right)$, where the elements of $\phi_{i}$ represent the parameters of the univariate GARCH model for the i−th CM series, $\phi_{i}=\alpha_{0i}$, $\alpha_{1i},\ldots ,\alpha_{p_{i}i}$, $\beta_{1i},\ldots ,\beta_{Q_{i}i}$. Based on equations (1) and (2) and DCC(1,1)-GARCH(1,1), n=1, m=1, we estimate a DCC(1,1)-GARCH(1,1) model:

(3) $$h_{1t}=\alpha_{01}+\alpha_{11}\varepsilon_{1,t-1}+\beta_{11}h_{1t-1}$$

(4) $$h_{2t}=\alpha_{02}+\alpha_{12}\varepsilon_{2,t-1}+\beta_{12}h_{2t-1}$$

(5) $$Q_{t}=(1-\alpha_{1}-\beta_{1})\bar{Q}+\alpha_{1}(\varepsilon_{t-1}\varepsilon_{t-1}^{\prime })+\beta_{1}Q_{t-1}$$

The Granger causality test was used to study return-volume correlations and examine the effect of the Covid Panic Index on the CMs returns.

## 3 Results and discussions

Our preliminary analysis begins with the correlation matrix for the ten pairs of CMs during the COVID-19 period. Table 2 reports the correlation estimates of the pairs of CMs and their significance. The correlation coefficients are positively significant for the pairs ADA-BTC, ADA-DOGE, ADA-DOT, ADA-ETH, ADA-XRP, BTC-DOGE, BTC-DOT, BTC-ETH, BTC-XRP, DOGE-DOT, DOGE-ETH, DOGE-XRP, DOT-XRP, DOT-ETH, ETH-XRP, LTC-RTX, LTC-XLM, TRX-XLM, and UNI-XLM. Unlike Imran and Shoaib (2020) [12], who found correlation above 0.620 for the pairs BTC-ETH, BTC-LTC, and ETH-LTC, lines (6-9) in Table 2 show that the correlation coefficients between the rest of the pairs are negatively significant and under –0.446. One might conclude at this level that the correlation estimates for CMs with negative coefficients of association indicate that these CMs serve during the COVID-19 crisis as hedge instruments against risky CMs with positive coefficients of association. In addition, we notice that the prices of almost all CMs decrease when PI increases.

**Table 2:** Conditional correlation GARCH model between pairs of CMs.

CM	ADA	BTC	DOGE	DOT	ETH	LTC	TRX	UNI	XLM	XRP	PI
ADA	1										
BTC	0.824^***^	1									
	[0.000]										
DOGE	0.809^***^	0.872^***^	1								
	[0.000]	[0.000]									
DOT	0.126*	0.090	0.124*	1							
	[0.053]	[0.174]	[0.059]								
ETH	0.864^***^	0.907^***^	0.887^***^	0.116*	1						
	[0.000]	[0.000]	[0.000]	[0.077]							
LTC	–0.076	–0.165^**^	–0.170^***^	–0.225^***^	–0.108*	1					
	[0.244]	[0.011]	[0.009]	[0.000]	[0.100]						
RTX	–0.376^***^	–0.429^***^	–0.446^***^	–0.036	–0.441^***^	0.206^***^	1				
	[0.000]	[0.000]	[0.000]	[0.583]	[0.000]	[0.001]					
UNI	–0.128^**^	–0.111*	–0.100	–0.065	–0.139^**^	–0.039	–0.056	1			
	[0.050]	[0.095]	[0.127]	[0.327]	[0.033]	[0.555]	[0.392]				
XLM	–0.178^***^	–0.154^**^	–0.143^**^	–0.138^**^	–0.173^***^	0.099	0.067	0.050	1		
	[0.006]	[0.018]	[0.028]	[0.034]	[0.008]	[0.127]	[0.307]	[0.449]			
XRP	0.835^***^	0.870^***^	0.842^***^	0.142^**^	0.913^***^	–0.137^**^	–0.426 ^***^	–0.110*	–0.156^**^	1	
	[0.000]	[0.000]	[0.000]	[0.030]	[0.000]	[0.036]	[0.000]	[0.093]	[0.016]		
PI	–0.079	–0.094	–0.130^**^	0.053	–0.114*	–0.015	0.079	0.058	-0.060	–0.104	1
	[0.226]	[0.150]	[0.046]	[0.419]	[0.080]	[0.810]	[0.228]	[0.378]	[0.359]	[0.113]	

Notes: Numbers below the matrix diagonal indicate correlation between pairs of CM. Numbers in brackets indicate their corresponding significance p-value. ^⁎⁎⁎^ (^⁎⁎^/^⁎^) denote rejection of the null hypothesis that parameter is equal to zero at 1% (5%/10%) significance level.

Source: Own calculations

**Figure 1:**
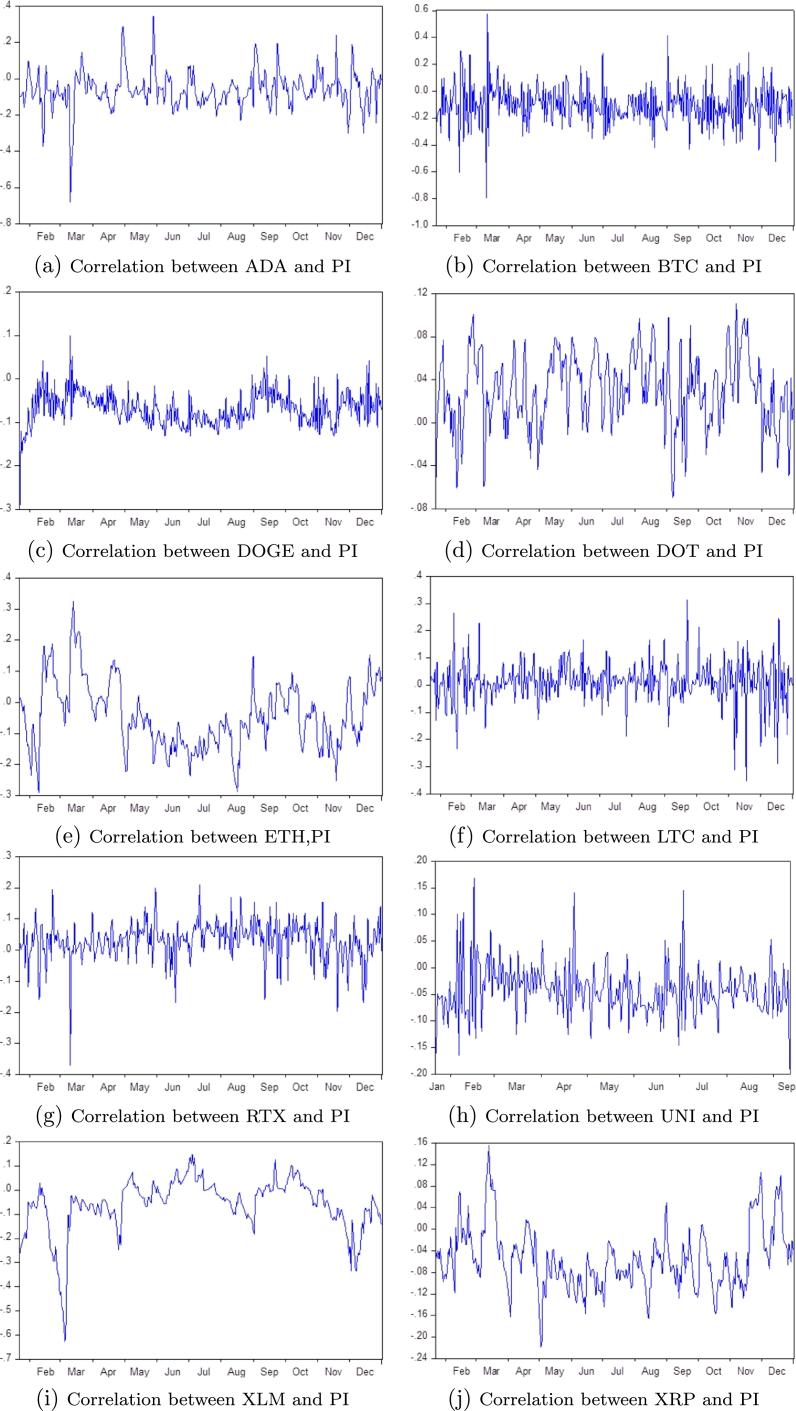
Dynamic conditional correlations between CMs and PI from January 1, 2020, to December 31, 2020.

To examine the CMs' returns and volatility of Cardano, Bitcoin, Dogecoin, Polkadot, Etherum, Litcoin, Tron, Uniswap, Stellar, and Ripple, we use the multivariate DCC-GARCH model written in equations (3)-(5). The results for the DCC(1,1)-GARCH(1,1) model are reported in Table 3. As shown in Table 3, the estimated parameters $\alpha_{11},\alpha_{12}$ are significantly positive, except those of ADA, ETH, and XRP, respectively, indicating that there are no ARCH effects for these three CMs. All estimated GARCH model parameters ($\beta_{11},\beta_{12}$) are statistically positive, indicating that there are significant autocorrelation and GARCH effects for the returns of all CMs. Conditional variance of CM returns are influenced by past innovations ($\alpha_{11},\alpha_{12}$) and by their lagged variances ($\beta_{11},\beta_{12}$). The time-varying correlations for each CM and PI are presented in Fig. 1. Fig. 1(a-j) indicates that the period is marked by a great moment of shocks, which appeared more when the WHO announced the pandemic as a health crisis in March 2020 [27], [28], [29]. Therefore, the CMs' gregarious behavior is detected [23]. The DCC parameters $\alpha_{1}$ and $\beta_{1}$ are statistically significant in all cases except those of both DOT and XLM. We also notice that $\beta_{1}>\alpha_{1}$ for all cases. We therefore argue that behavior of current variances is more affected by the magnitude of past variances than by past innovations. The sum of DCC parameters ($\alpha_{1}+\beta_{1}$) is larger than zero, indicating that conditional correlation between CMs and PINDEX is not constant.

**Table 3:** Results of the DCC(1,1)–GARCH(1,1) estimates: PI index and CMs returns.

CM	*α*_01_	*α*_02_	*α*_11_	*α*_12_	*β*_11_	*β*_12_	*α*_1_	*β*_1_	LM test
ADA	0.0009	0.1583^⁎⁎^	0.06047	0.1904^⁎⁎^	0.7257^⁎⁎⁎^	0.4926^⁎⁎⁎^	0.1073^⁎⁎^	0.5979^⁎⁎⁎^	0.00003
	[0.207]	[0.011]	[0.144]	[0.019]	[0.000]	[0.006]	[0.020]	[0.000]	[0.995]
BTC	0.0001^⁎⁎⁎^	0.1763^⁎⁎⁎^	0.2087^⁎⁎⁎^	0.1781^⁎⁎^	0.8136^⁎⁎⁎^	0.4706^⁎⁎⁎^	0.1928^⁎⁎⁎^	0.6188^⁎⁎⁎^	0.00762
	[0.001]	[0.004]	[0.000]	[0.024]	[0.000]	[0.007]	[0.000]	[0.000]	[0.930]
DOGE	0.0001^⁎⁎^	0.2530^⁎⁎⁎^	0.2334^⁎⁎⁎^	0.1352^⁎^	0.8006^⁎⁎⁎^	0.3412^⁎^	0.1776^⁎⁎⁎^	0.5227^⁎⁎⁎^	1.88331
	[0.029]	[0.002]	[0.000]	[0.052]	[0.000]	[0.077]	[0.000]	[0.000]	[0.170]
DOT	0.0005^⁎^	0.1310^⁎⁎⁎^	0.0939^⁎^	0.4423^⁎⁎⁎^	0.7831^⁎⁎⁎^	0.4252^⁎⁎⁎^	0.0154	0.86494	0.00195
	[0.071]	[0.004]	[0.060]	[0.004]	[0.000]	[0.001]	[0.832]	[0.200]	[0.964]
ETH	0.0004	0.1687^⁎⁎^	0.0467	0.3289^⁎⁎^	0.7985^⁎⁎⁎^	0.4429^⁎⁎⁎^	0.1239^⁎⁎^	0.5947^⁎⁎⁎^	0.18304
	[0.122]	[0.010]	[0.115]	[0.013]	[0.000]	[0.005]	[0.019]	[0.000]	[0.668]
LTC	0.0001^⁎⁎⁎^	0.2227^⁎⁎⁎^	0.1464^⁎⁎⁎^	0.2113^⁎⁎^	0.8491^⁎⁎⁎^	0.3445^⁎⁎⁎^	0.1758^⁎⁎⁎^	0.5408^⁎⁎⁎^	0.27774
	[0.003]	[0.000]	[0.000]	[0.028]	[0.000]	[0.000]	[0.000]	[0.000]	[0.598]
TRX	0.0000^⁎⁎⁎^	0.1486^⁎⁎^	0.1429^⁎⁎⁎^	0.1465^⁎⁎^	0.8768^⁎⁎⁎^	0.5512^⁎⁎⁎^	0.1447^⁎⁎⁎^	0.6952^⁎⁎⁎^	1.67341
	[0.000]	[0.020]	[0.000]	[0.015]	[0.000]	[0.001]	[0.000]	[0.000]	[0.195]
UNI	0.0000	0.2179^⁎⁎⁎^	0.2268^⁎⁎⁎^	0.2140^⁎⁎^	0.7958^⁎⁎⁎^	0.3166^⁎^	0.2230^⁎⁎⁎^	0.5020^⁎⁎⁎^	0.96360
	[0.118]	[0.006]	[0.004]	[0.048]	[0.000]	[0.100]	[0.002]	[0.001]	[0.308]
XLM	0.0005^⁎⁎^	0.1438^⁎⁎⁎^	0.1727^⁎⁎⁎^	0.4544^⁎⁎⁎^	0.6805^⁎⁎⁎^	0.4173^⁎⁎⁎^	0.14709	0.1510	1.11118
	[0.016]	[0.005]	[0.004]	[0.007]	[0.000]	[0.002]	[0.299]	[0.816]	[0.291]
XRP	0.0003^⁎⁎⁎^	0.1375	0.3316^⁎⁎⁎^	0.0521	0.6014^⁎⁎⁎^	0.6628^⁎⁎⁎^	0.1314^⁎⁎^	0.6628^⁎⁎⁎^	0.00905
	[0.003]	[0.327]	[0.000]	[0.230]	[0.000]	[0.000]	[0.034]	[0.000]	[0.702]

Notes: The table reports the estimates of the GARCH and DCC-GARCH models described in Eq.(8-10). In parentheses under the parameter estimation, p-values are given. ^⁎⁎⁎^ (^⁎⁎^/^⁎^) denote rejection of the null hypothesis that parameter is equal to zero at 1% (5%/10%) significance level. LM test is a test for ARCH disturbance. The time span is: January 1 through December 31, 2020.

Source: Own calculations

**Figure 2:**
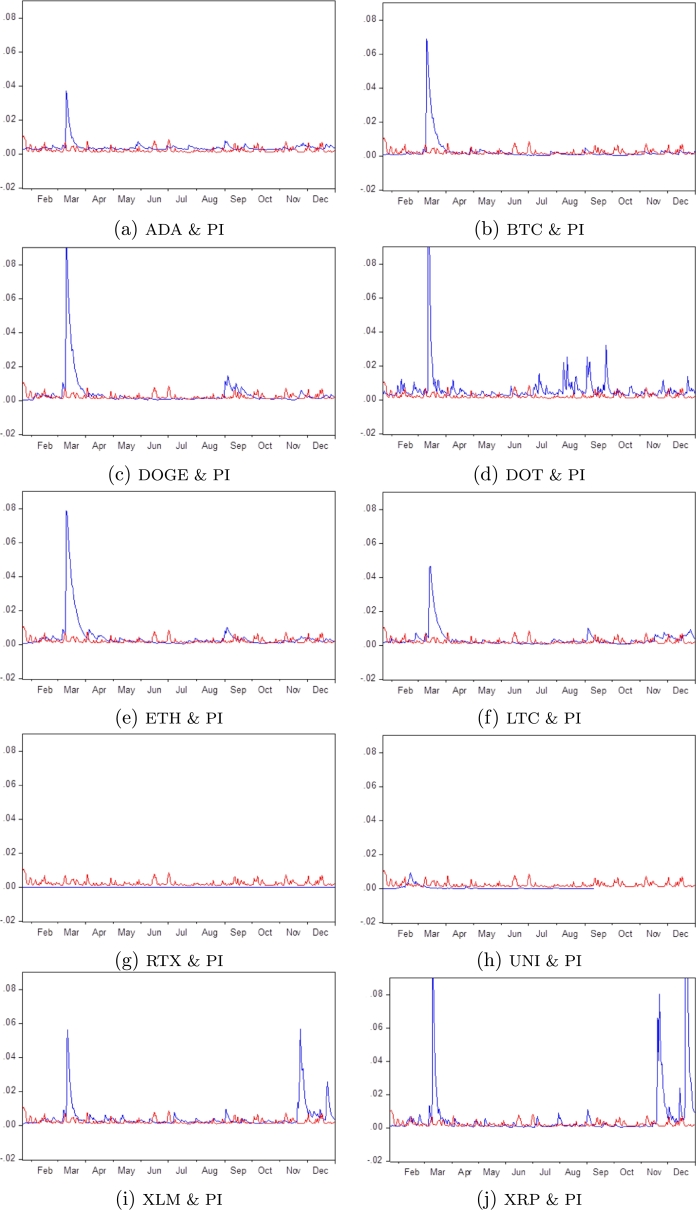
CMs (line blue) and PI (line red) conditional volatility from January 1, 2020, to December 31, 2020.

Another exciting finding provided in Table 3 shows that 7 out of the 10 CMs or 70% volatility is very sensitive to the COVID-19 shocks ($\alpha_{11}>0.1$). These are BTC, DOGE, LTC, TRX, UNI, XLM, and XRP. In contrast, ADA, DOT, and ETH have volatility that seems less affected by COVID-19 shocks. For persistence, we notice that all CMs, it tends to decrease rapidly as a result of the COVID-19 crisis ($\beta_{11}<0.9$). Finally, we note that the rate of convergence ($\alpha_{11}+\beta_{11}$) of volatility towards the average is high for all CMs at around 1. This finding means volatility stability converges towards its average in the long-term (cf. Fig. 2(a-j)). The results of the Lagrange Multiplier (LM) test confirm the stability conditions of the used models, suggesting that a DCC(1,1)-GARCH(1,1) model is appropriately specified. The correlation between Pindex and CMs yield varies over time, which is in line with the conclusions of [11], [3], [9]. Moreover, there is a strong and time-varying relationship between the volatility of the return and the pindex. A contagion effect is marked (Fang et al., 2022). Table 4 demonstrates a less than 5% probability for six CMs, meaning there is Granger causality. This finding indicates that the effect of the Covid-19 crisis is only significant for large and Known cryptocurrencies, which supports our main results.

**Table 4:** Granger causality test.

Pindex granger to	Obs.	F–statistic	Prob.	Causality
RADA	343	3.325	0.037	Yes
RBTC	343	5.983	0.00	Yes
RDOGE	343	6.587	0.002	Yes
RDOT	343	5.105	0.007	Yes
RETH	343	3.623	0.028	Yes
RLTC	343	0.319	0.728	No
RTRX	343	2.277	0.104	No
RUNI	343	0.911	0.404	No
RXLM	343	6.591	0.002	No
RXRP	343	0.993	0.371	Yes

The Covid-19 crisis has significantly impacted the returns of cryptocurrencies (CM). At the beginning of the pandemic, traditional financial markets experienced a sharp decline, which led some investors to turn to alternative assets such as CM in the hope of protecting their portfolios. In addition, the health crisis has also stimulated the adoption of blockchain technology, which is the basis of most cryptocurrencies. With confinement, online exchanges have multiplied, and the demand for faster and more secure transactions has increased. The blockchain offers these advantages and has therefore experienced strong growth during the crisis. But it is important to note that the returns of CM remain highly volatile and can vary considerably depending on economic and geopolitical events.

## 4 Conclusion and limitations

This study used a DCC-GARCH model to determine the impact of the coronavirus index on ten significant cryptocurrency returns during the COVID-19 pandemic. The econometric results show that 7 out of the 10 CMs or 70% volatility is very sensitive to the COVID-19 shocks ($\alpha_{11}>0.1$). These are BTC, DOGE, LTC, TRX, UNI, XLM, and XRP. In contrast, ADA, DOT, and ETH have volatility that seems less affected by COVID-19 shocks. For persistence, we notice that all CMs tend to decrease rapidly as a result of the COVID-19 crisis ($\beta_{11}<0.9$).

Studying the impact of COVID-19 on cryptocurrency markets is very relevant and has many benefits. Indeed, this study should be left to investors and portfolio managers who actively deal with Cardano, Bitcoin, Dogecoin, Polkadot, Etherum, Litcoin, Tron, Uniswap, Stellar, and Ripple. However, any change in BTC, DOGE, LTC, TRX, UNI, XLM, and XRP requires careful follow-up from policymakers if they want to avoid adverse consequences from contagious shocks. In addition, the outcomes can help investors develop hedging strategies appropriate to the risk and investment objectives. For example, since cryptocurrencies can be volatile, investors may need to hedge against potential losses. The insights from this study into the risk-return characteristics of cryptocurrencies can help investors make informed decisions about their portfolio allocation.

There are notable limitations to this study. First, it covers only the period from January 1 to December 31, 2020, which may only represent part of the COVID-19 pandemic period. Future research could extend the analysis to cover a longer period, including before and after the pandemic. Second, the sample size used in this study is limited to ten major cryptocurrencies, which may represent only some of the cryptocurrency market. Future research could include a larger sample of cryptocurrencies to improve the generalizability of the findings. Third, this study needs to consider the highly divergent contexts in the countries. Future research could investigate whether the impact of COVID-19 on cryptocurrency returns and volatility varies across different countries or regions, as the pandemic's severity and government policies differ.

## CRediT authorship contribution statement

Kais Ben-Ahmed conceived and designed the analysis.

Kais Ben-Ahmed & Saliha Theiri analyzed and interpreted the data.

Saliha Theiri & Naziha Kasraoui: Contributed analysis tools or data.

Saliha Theiri & Naziha Kasraoui: Contributed reagents, materials, analysis tools or data.

Kais Ben-Ahmed wrote the paper.

## Declaration of Competing Interest

The authors declare that they have no known competing financial interests or personal relationships that could have appeared to influence the work reported in this paper.

## Data Availability

Data will be made available on request.

## References

[1] Apergis N. (2021). COVID-19 and cryptocurrency volatility: evidence from asymmetric modelling. Finance Res. Lett..

[2] Bouri E., Roubaud D., Shahzad S.J.H. (2019). Do Bitcoin and other cryptocurrencies jump together?. Q. Rev. Econ. Finance.

[3] Batten J.A., Choudhury T., Kinateder H., Wagner F.N. (2022). Volatility impacts on the European banking sector: GFC and COVID-19. Ann. Oper. Res..

[4] Chiang T.C., Jeon B.N., Li H. (2007). Dynamic correlation analysis of financial contagion: evidence from Asian markets.

[5] Corbet S., Larkin C., Lucey B. (2020). The contagion effects of the COVID-19 pandemic: evidence from gold and cryptocurrencies. Finance Res. Lett..

[6] Cho J.H., Parhizgari A.M. (2009). East Asian financial contagion under DCC-Garch. Int. J. Bank. Finance.

[7] Charfeddine L., Benlagha N., Maouchi Y. (2020). Investigating the dynamic relationship between cryptocurrencies and conventional assets: implications for financial investors. Econ. Model..

[8] Forbes K., Rigobon R. (2002). No contagion, only interdependence: measuring stock market comovements. J. Finance.

[9] Fang F., Ventre C., Basios M. (2022). Cryptocurrency trading: a comprehensive survey. Financ. Innov..

[10] Goodell J.W. (2020). COVID-19 and finance: agendas for future research. Finance Res. Lett..

[11] Gradojevic N., Tsiakas I. (2021). Volatility cascades in cryptocurrency trading. J. Empir. Finance.

[12] Imran Y., Shoaib A. (2020). The COVID-19 outbreak and high frequency information transmission between major cryptocurrencies: evidence from the VAR-DCC-GARCH approach. Borsa Istanb. Rev..

[13] Karaka Y., Baleanu D. (2020). A novel R/S fractal analysis and wavelet entropy characterization approach for robust forecasting based on self-similar time series modelling. Fractals.

[14] Karamti C., Belhssine O. (2021). COVID-19 pandemic waves and global financial markets: evidence from wavelet coherence analysis. Finance Res. Lett..

[15] Liu Y., Tsyvinski A. (2018).

[16] Liu J., Serletis A. (2019). Volatility in the cryptocurrency market. Open Econ. Rev..

[17] Lahmiri S., Bekiros S. (2019). Decomposing the persistence structure of Islamic and green crypto-currencies with nonlinear stepwise filtering. Chaos Solitons Fractals.

[18] Lahmiri S., Bekiros S. (2018). Time-varying self-similarity in alternative investments. Chaos Solitons Fractals.

[19] Lahmiri S., Bekiros S., Bezzina F. (2020). Multi-fluctuation nonlinear patterns of European financial markets based on adaptive filtering with application to family business, green, Islamic, common stocks, and comparison with Bitcoin, NASDAQ, and VIX. Chaos Solitons Fractals.

[20] Lahmiri S., Stelios B.S. (2021). The effect of COVID-19 on long memory in returns and volatility of cryptocurrency and stock markets. Chaos Solitons Fractals.

[21] Naeem N., Shahbaz M., Saleem K., Mustafa F. (2019). Risk analysis of high frequency precious metals returns by using long memory model. Resour. Policy.

[22] Oprean C., Tanasescu C. (2014). Fractality evidence and long-range dependence on capital markets: a Hurst exponent evaluation. Fractals.

[23] Ozdemir O. (2022). Cue the volatility spillover in the cryptocurrency markets during the COVID-19 pandemic: evidence from DCC-GARCH and wavelet analysis. Financ. Innov..

[24] Stosic T., Abarghouei Nejad S., Stosic B. (2020). Multifractal analysis of Brazilian agricultural market. Fractals.

[25] Umar Z., Gubareva M. (2020). A time–frequency analysis of the impact of the Covid-19 induced panic on the volatility of currency and cryptocurrency markets. J. Behav. Exp. Finance.

[26] Vidal-Tomas D. (2021). Transitions in the cryptocurrency market during the COVID-19 pandemic: a network analysis. Finance Res. Lett..

[27] WHO (March 2020). Director-General's opening remarks at the Media Briefing on COVID-19-11. https://www.who.int/dg/speeches/detail/who-director-generals-opening-remarks-at-the-media-briefing-on-covid-19---11-march-2020.

[28] WHO (2020). Coronavirus disease 2019 (COVID-19). https://www.who.int/docs/default-source/coronaviruse/situation-reports/20200316-sitrep-56-covid-19.pdf?sfvrsn=9fda7db2-6.

[29] WHO (2020-03-25). Coronavirus disease 2019 (COVID-19). https://www.who.int/docs/default-source/coronaviruse/situation-reports/20200324-sitrep-64-covid-19.pdf?sfvrsn=703b2c40-2.

[30] Zhang Y., Chan S., Chu J., Nadarajah S. (2019). Stylised facts for high frequency cryptocurrency data. Physica A.

[31] Zhang D., Hu M., Ji Q. (2020). Financial markets under the global pandemic of COVID-19. Finance Res. Lett..

